# Optimized Gingiva Cell Behavior on Dental Zirconia as a Result of Atmospheric Argon Plasma Activation

**DOI:** 10.3390/ma16124203

**Published:** 2023-06-06

**Authors:** Susanne Staehlke, Jakob Brief, Volkmar Senz, Thomas Eickner, J. Barbara Nebe

**Affiliations:** 1 Institute for Cell Biology, Rostock University Medical Center, 18057 Rostock, Germany; barbara.nebe@med.uni-rostock.de; 2 VITA Zahnfabrik H. Rauter GmbH & Co. KG, 79713 Bad Säckingen, Germany; j.brief@vita-zahnfabrik.com; 3 Institute for Biomedical Engineering, Rostock University Medical Center, 18119 Rostock, Germany; volkmar.senz@uni-rostock.de (V.S.); thomas.eickner@uni-rostock.de (T.E.); 4 Department Science and Technology of Life, Light and Matter, University of Rostock, 18059 Rostock, Germany

**Keywords:** zirconium, cold atmospheric pressure plasma, scanning electron microscopy, XPS, human gingival cells, cell morphology, actin cytoskeleton, spreading, ATP receptors, Ca^2+^ signaling

## Abstract

Several physico-chemical modifications have been developed to improve cell contact with prosthetic oral implant surfaces. The activation with non-thermal plasmas was one option. Previous studies found that gingiva fibroblasts on laser-microstructured ceramics were hindered in their migration into cavities. However, after argon (Ar) plasma activation, the cells concentrated in and around the niches. The change in surface properties of zirconia and, subsequently, the effect on cell behavior is unclear. In this study, polished zirconia discs were activated by atmospheric pressure Ar plasma using the kINPen^®^09 jet for 1 min. Surfaces were characterized by scanning electron microscopy, X-ray photoelectron spectroscopy (XPS), and water contact angle. In vitro studies with human gingival fibroblasts (HGF-1) focused on spreading, actin cytoskeleton organization, and calcium ion signaling within 24 h. After Ar plasma activation, surfaces were more hydrophilic. XPS revealed decreased carbon and increased oxygen, zirconia, and yttrium content after Ar plasma. The Ar plasma activation boosted the spreading (2 h), and HGF-1 cells formed strong actin filaments with pronounced lamellipodia. Interestingly, the cells’ calcium ion signaling was also promoted. Therefore, argon plasma activation of zirconia seems to be a valuable tool to bioactivate the surface for optimal surface occupation by cells and active cell signaling.

## 1. Introduction

Dental implants are utilized to support prosthetic rehabilitation, and replace missing teeth [1,2]. In the last decades, replacements have become increasingly common, so scientific research is constantly developing endosseous dental implants, improving the material surface quality [1,2,3]. One of the most studied alternative materials to titanium in the dental field is bioinert zirconia (ZrO_2_), which is biocompatible with soft and hard tissues and satisfies functional as well as aesthetic requirements [2,4,5,6,7,8].

Oral implant materials must have optimal surface compatibility with bone and soft tissue and antimicrobial properties on an exposed area of the mucosa [5,9]. Among other things, soft tissue adhesion to the transmucosal parts of an implant (abutments) plays a crucial role in the long-term success of dental implants [10]. Soft tissue sealing [11,12] is very significant because the abutment is in direct contact with the junctional epithelium and connective tissue (fibroblasts), which serve as a protective barrier for the underlying bone [8]. To achieve this soft tissue seal, human gingival fibroblasts (HGFs), considered the most critical cell type in peri-implant soft tissue [13], must adhere to the surface before bacteria so that a layer of cells can cover the abutment [14]. Comparing different dental materials, fibroblast viability and proliferation were higher on polyetheretherketone and yttria-stabilized zirconia groups than on titanium (Ti) [15].

The integration of soft but also hard tissue in modern dental implants is mainly determined by the topographical, biological, and chemical surface conditions [1,16,17]. In recent years, modifications of surface topography [18,19,20] and chemistry have been introduced to optimize oral implants to increase bioactivity [21,22].

The surface modification with physical plasma has become the focus of in vitro research. In a review on this topic, Carossa et al. [10] concluded that due to plasma activation, faster cell adhesion of murine or human fibroblasts and protein adsorption in the earliest times (hours) could be reached. This highlighted the potential benefits of physical plasma treatment of titanium bony fixtures and abutments [10]. Fewer studies were found and cited concerning zirconia materials and plasma activation.

Physical plasma, the so-called fourth state of matter, is generated when energy continues to be added to a gas. An electrically neutral ionized gas produces a mixture of reactive oxygen and nitrogen species (RONS), excited molecules, charged particles, chemically reactive neutral particles, and UV radiation [23,24,25]. The kINPen (INP e.V. Greifswald) is a cold atmospheric pressure plasma jet where the plasma is discharged in the inert argon (Ar) gas [23,26]. Considering the low application temperature, there is no damage to the material and acceptable thermal tissue damage [27], suggesting atmospheric pressure plasma is a promising device in clinical practice [28,29]. Various interactions between plasma and a material surface are described [30]. Protein adsorption and subsequent cell behavior depend on surface wettability and electrical charge [10,31,32], which can be modified by physical plasma treatment. Physico-chemical surface properties of materials control the complex cellular behavior [33,34] by transduction of the external stimuli and forces into intracellular biochemical signals [35]. Adhesion receptors such as integrins facilitate subsequent signal transduction across the actin cytoskeleton [36]. Furthermore, intracellular calcium ions (Ca^2+^) as second messengers [37] act on various cellular processes, such as the regulation of cytoskeletal components [38] or proliferation.

Guo et al. [35] found out that fibroblasts (L929, HGFs) on oxygen plasma and also ultraviolet (UV)-light-treated surfaces extended most not only on machined titanium and polyether-ether-ketone (PEEK) but also on zirconia surfaces. Further studies investigated the (i) response of fibroblasts on biomaterial surfaces functionalized with Ar plasma [39] or the (ii) reaction of osteoblast-like cells to surfaces activated with Ar or oxygen (O) plasma [31,40,41]. A direct comparison of Ar- and O_2_-activated biomaterials revealed that the influence of Ar plasma was less pronounced for the adhesion and proliferation of osteoblasts and gingival fibroblasts [35,41].

In previous studies, we have shown that a topographical modification of ZrO_2_ with innovative sinusoidal structures positively affected cellular adhesion [20]. However, very rough surfaces or deep cavities hindered cellular attachment and the spreading of gingival fibroblasts [20,22]. We discovered that zirconia surfaces developed hydrophobic surface characteristics after laser-induced micro-roughening, which reduced cell spreading [22]. Additional surface activation with an Ar plasma jet only for 1 min could equalize this effect. The zirconia surfaces switched to the hydrophilic state with a higher surface free energy. As a result, the cells spread out faster and, astonishingly, could find a niche in the laser-induced holes and grooves [22].

Since faster cell spreading indicates higher cell activity, we are interested in whether cell signaling, e.g., Ca^2+^ dynamics, is also increased on a planar zirconia surface activated with argon plasma. Several studies have shown that Ca^2+^ dynamics as a sensitive parameter can reflect the physiological features of cells, such as actin cytoskeleton organization [42] and proliferation [43,44], depending on the growth on different materials. To our knowledge, no literature exists on intracellular Ca^2+^ dynamics in the context of atmospheric plasma modification of zirconia. The underlying mechanisms by which the surface is materially and biologically activated by cold plasma still need to be fully understood.

The present study aimed to evaluate in more detail the effects of atmospheric pressure argon plasma (ZrO_2_ + Ar) activation of polished dental zirconia on surface properties and subsequently on interactions with HGF-1 soft tissue cells in terms of cell attachment, morphology, cell viability, and Ca^2+^ signaling within 24 h. These cell parameters are essential during the initial interaction between the cell and the biomaterial interface [43].

## 2. Materials and Methods

### 2.1. Zirconia Specimens and Argon (Ar) Plasma Activation

As bulk material, polished Yttria-stabilized zirconia discs were used with a diameter of 12 mm and a thickness of 1.5 mm (**ZrO_2_**) [22].

To modulate the wettability, the zirconia specimens were activated by a cold atmospheric pressure plasma jet kINPen^®^09 (Neoplas Tools GmbH, Greifswald, Germany) [22,23,45] (**ZrO_2_ + Ar**). The argon plasma source consists of a quartz capillary with a high-frequency (HF) electrode to which an HF voltage of 1.1 MHz/2–6 kV was applied. The settings at the power supply were 60.0 V and 0.05 A. The gas flow of argon (99.99%) was 1.9 slm. To activate the surfaces, the plasma jet meandered perpendicularly with the quartz capillary 1 cm above the surface for 60 s so that the plasma was in direct contact with the entire surface.

For further control, glass substrates with a collagen type I coating were used [33] (**COL**). Collagen type I is used as a biological matrix because it is the most abundant extracellular matrix protein in mammals and provides a biocompatible cell environment [46]. For coating, collagen type I (rat tail, 3.32 mg/mL, BD Biosciences, Heidelberg, Germany) was diluted in 0.1% acetic acid (CH_3_COOH; Sigma-Aldrich, Munich, Germany) to 200 µg/mL. The working solution was dropped onto the glass substrates (Ø 12 mm, Menzel GmbH, Braunschweig, Germany) and dry overnight under sterile conditions in a laminar flow box. Afterward, the specimens were rinsed three times with phosphate buffer solution (PBS, Sigma-Aldrich).

### 2.2. Chemical Surface Properties

*Wettability of surfaces:* A sessile drop method by Drop Shape Analyzer DSA25 (Krüss, Hamburg, Germany) [22] was used for the measurement of the water contact angle (WCA) of the substrate/air interface. Therefore, one drop with 1 µL of distilled water was deposited onto the material surface. Three to five drops per specimen were measured due to the hydrophilicity of the materials. Using the digital camera of the DSA25 and the supplied software (ADVANCE, V.1.7.2.1, Krüss, Hamburg, Germany), drop images were acquired, and wettability values were evaluated with the optimal fit method (ellipse, tangent, circle, height/width manual) according to the curvature of the drop shape.

*X-ray photoelectron spectroscopy (XPS):* Surface chemistry of two samples, one following plasma treatment (ZrO_2_ + Ar) and one reference sample (ZrO_2_), was investigated using X-ray Photoelectron Spectroscopy (K-Alpha XPS System, Thermo Fisher Scientific GmbH, Dreieich, Germany) using the Al-Kα line at 1486.6 eV. Survey scans and detailed spectra of prominent characteristic peaks (C1s, O1s, Si2p, Zr3d, Y3d) were taken on 9 points per sample, i.e., a 3 × 3-point grid, to reduce local charging and account for possible surface inhomogeneities and, finally, summed up (pass energy: 30 eV, energy step size: 100 meV, dwell time: 0.1 s, number of scans: 10, spot size: 400 µm for detailed scans).

### 2.3. Gingival Cell Culture and Characterization

*Cell culture:* Human gingival fibroblasts (HGF-1, ATCC^®^, CRL-2014™, Manassas, VA, USA) were used in these experiments as they are essential for maintaining, wound healing, and regenerating gingival connective tissue [13]. HGF-1 cells were cultured in Dulbecco’s modified Eagle’s medium (DMEM; high glucose, GlutaMAX; Thermo Fisher Scientific, Gibco, Paisley, UK) with 10% fetal bovine serum (FBS Premium, South American origin; PAN Biotech, Aidenbach, Germany), and 1% antibiotic-antimycotic (penicillin, streptomycin, and amphotericin B; Anti-Anti 100×, Thermo Fisher Scientific, Gibco) (represents complete medium) at 37 °C and 5% CO_2_. The number of cells required for the experiments was seeded in a meandering pattern on the specimens.

*Cell adhesion and growth over time*: To study the adhesion, spreading, and growth of HGF-1 gingival fibroblasts, cells were cultured on tissue culture polystyrene (TCPS) for up to seven days (168 h) and observed at specific intervals by phase contrast microscope (Axiovert 40, Carl Zeiss, Oberkochen, Germany). The AxioVision software (Axio vs. 40 × 64 V 4.9.1.0; Zeiss) and camera (AxioCam CC1; Zeiss) were used for documentation.

*Adhesion receptors*: To further characterize the HGF-1 gingival fibroblasts, the expression of adhesion receptors was analyzed in the cells. For this purpose, cells were washed twice with PBS, trypsinized with trypsin/ethylenediaminetetraacetic acid (0.25% trypsin/0.38% EDTA; Invitrogen, Gibco, Paisley, UK) for 5 min, stopped by the addition of complete medium, and centrifuged at 1200 rpm for 5 min in FACS tubes (BD, á 50,000 cells). Cell pellets were then incubated with primary monoclonal anti-integrin antibodies at room temperature (RT) for 30 min: CD44 (1:100, mouse), α2 (1:33, mouse), α3 (1:40, mouse), α5 (1:10, mouse), αV (1:160, goat), β1 (1:25, mouse), β3 (1: 20, mouse, FITC) (all from Thermo Fisher Scientific, diluted in PBS), or for control with mouse IgG1 (1:10; BD Biosciences, Heidelberg, Germany). After washing step, cells were centrifuged (1800 rpm for 5 min), and the pellet incubated in secondary anti-mouse IgG antibody (whole molecule F(ab’) fragment-FITC (1:15) for CD44, α2, α3, α5, β1 (Sigma-Aldrich), or donkey anti-goat Alexa Fluor 488 (1:100, Thermo Fisher Scientific) for αV in the dark for 30 min. Following a final wash and centrifugation step, the pellet was resuspended in Cellfix (BD, 1:10). Then, 10,000 events were acquired on the flow cytometer FACSCalibur (Becton Dickinson, BD Biosciences) equipped with an argon-ion laser (λ 488 nm) and CellQuestPro software (4.0.1; BD Biosciences). FL1-H (for FITC) was analyzed as the mean channel (arbitrary units of fluorescence intensity) using FlowJo_V.10.1r1 (FlowJo, LLC; BD Becton Dickinson and Company, Franklin Lakes, NJ, USA).

*Adenosine 5’-triphosphate (ATP) receptors*: In the pre-screening study, we also analyzed the expression of ATP receptors in gingival fibroblasts. Therefore, HGF-1 cells (50,000 cells/cm^2^) were cultured on ibidi µ-dish (ibiTreat, Ø 35 mm; ibidi GmbH, Martinsried, Germany). After 24 h, cells were fixed with 4% paraformaldehyde (PFA, Sigma-Aldrich Chemie, Taufkirchen, Germany) and permeabilized with 0.1% Triton X-100 (10 min, RT) (Merck, Darmstadt, Germany) even at RT for 10 min. ATP receptors were stained with the primary antibodies anti-P2Y2 (1:100, Alomone Labs, Jerusalem, Israel) and anti-P2X7 (1:200, Alomone Labs) at RT for 60 min. After washing, cells were labeled with goat-anti-rabbit-IgG-Alexa Fluor 488 (1:200, Invitrogen) at RT in the dark for 30 min. In the end, HGF-1 cells were embedded with Fluoroshield^TM^ with 4′,6-diamidino-2-phenylindole (DAPI, Sigma-Aldrich) and stored in the dark at 4 °C [32]. The expression of receptors was observed by LSM780 (Carl Zeiss) with a Plan-Apochromat 63×/1.40 Oil DIC M27 objective (Carl Zeiss) and the ZEN software (ZEISS efficient navigation, ZEN 2011 SP4, black and blue editions, Carl Zeiss).

### 2.4. Cell Morphology and Actin Cytoskeleton Organization

HGF-1 gingival fibroblasts were grown on specimens for 2 h (70,000 cells/specimen) and 24 h (50,000 cells/specimen) at 37 °C. To analyze the cell morphology, HGF-1 cells were fixed with 2.5% glutaraldehyde (GA, Merck, Darmstadt, Germany). After dehydration through an ascending ethanol series (30, 50, 75, 90, and 100%), the specimens were dried in the K850 critical point dryer (Emitech, Taunusstein, Germany) and finally vaporized with carbon (EM SCD 500, Co. Leica, Bensheim, Germany) [22]. To illustrate the cells, a field emission scanning electron microscopy (FE-SEM, 5 kV; Merlin VP compact, Carl Zeiss) was equipped with a high-efficiency secondary electron detector (HE-SE) and an InlensDuo detector.

For actin cytoskeleton staining, HGF-1 cells were washed with PBS, fixed with 4% PFA, and further permeabilized with 0.1% Triton X-100 at RT for 10 min, respectively. Afterward, cells were incubated with phalloidine tetramethyl-rhodamine (TRITC, 1:15 in PBS, Sigma Aldrich) at RT in the dark for 30 min. In the final step, the HGF-1 cells were embedded with Fluoroshield^TM^ with DAPI and the organization of the actin cytoskeleton was recorded on the confocal laser scanning microscope LSM 780 (see Section 2.3).

### 2.5. Cell Spreading

To calculate the cell areas of HGF-1 cells on specimens after 2 and 24 h, the LSM images of the actin fluorescence were analyzed by ImageJ (Version 1.51f, Wayne Rasband, National Institutes of Health, Bethesda, MD, USA). Therefore, the pixels were converted into µm, and 40 cells/specimen were manually marked to compute the cell area (µm^2^).

### 2.6. Calcium Ion (Ca^2+^) Mobilization

To investigate the intracellular Ca^2+^ dynamics of HGF-1 gingival fibroblasts in dependence on plasma activation of zirconia, 100,000 cells/specimen were grown for 24 h, rinsed with PBS, and then stained in hypotonic 4-(2-hydroxyethyl)-1-piperazineethanesulfonic acid buffer (HEPES) with the calcium indicator Fluo-3/acetoxymethyl ester (AM) (5 μM, Life Technologies Corporation, Eugene, OR, United States) for 40 min at 37 °C. Our method was described elsewhere [46,47]. For analysis on the inverse LSM780, specimens were placed in an IBIDI μ-dish with the adherent cells facing the bottom and covered with isotonic HEPES buffer. A 40× C-Apochromat (1.2 W Korr M27) objective (Carl Zeiss) was used for the global calcium signal of the vital HGF-1 cells. The Fluo-3/AM calcium indicator was excited with the argon ion laser at 488 nm (emission at 515 nm), and the fluorescence signal was analyzed with Zen2011 (black edition) software (Carl Zeiss) and “time series” mode (time series included 240 cycles every 2 s). After the 90th cycle, HGF-1 cells were stimulated with 10 μM ATP (SERVA Electrophoresis GmbH, Heidelberg, Germany) to trigger a release of Ca^2+^ from the endoplasmic reticulum (intracellular Ca^2+^ store). The mean fluorescence intensity (MFI) of the fluorescence signal was analyzed using Zen2012 (blue edition) software and Mean ROI mode for defined ranges of single cells (n = 10 single cells per independent experiment) [32,42].

### 2.7. Statistical Analysis

The specimen size *n* consisted of at least three biologically independent replicates in each experiment (indicated in the corresponding figure). The software GraphPad Prism Version 7.02 (GraphPad Software Inc., La Jolla, CA, USA) was used for statistical evaluation and graph generation. Data were presented as median ± interquartile range (IQR for WCA graph) or mean ± standard error of the mean (s.e.m.). Group differences were investigated by analysis of variance. According to the assumption of normal distribution (Shapiro–Wilk normality test) and homoscedasticity (Bartlett’s test for equal variances of k samples) of the data either a parametric one-way ANOVA with post hoc Bonferroni test (for Ca^2+^ mobilization data set at 240 and 242 s) or a nonparametric Kruskal–Wallis test post tested pairwise with Mann–Whitney U test using a Bonferroni-adjusted significance level (for cell spreading, WCA, Ca^2+^ mobilization data) was performed. A *p*-value (* *p* ≤ 0.05) indicated statistical significance.

## 3. Results

### 3.1. Characterization of Chemical Surface Properties

**Figure 1:**
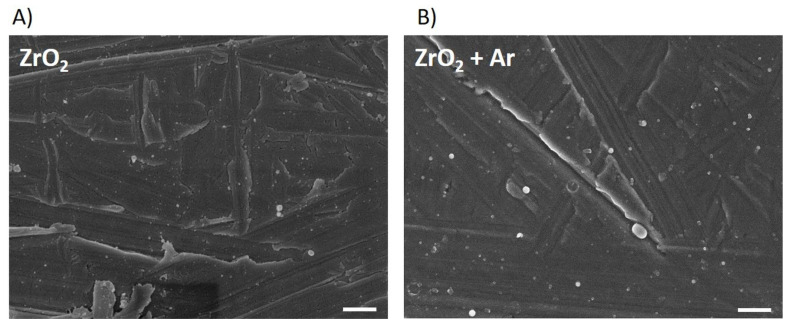
Zirconia specimen without (**A**) and after Ar plasma activation (**B**). Note that no structural differences are visible. (FE-SEM Merlin compact, Carl Zeiss; 5 kV, InlensDuo, 20,000×, scale bars = 500 nm).

The polished zirconia (ZrO_2_) indicated slightly grinding grooves without any orientation, as seen in FE-SEM images (Figure 1A). The Ar plasma activation (ZrO_2_ + Ar) did not exhibit any visible changes in surface topology in the nm range (Figure 1B).

**Figure 2:**
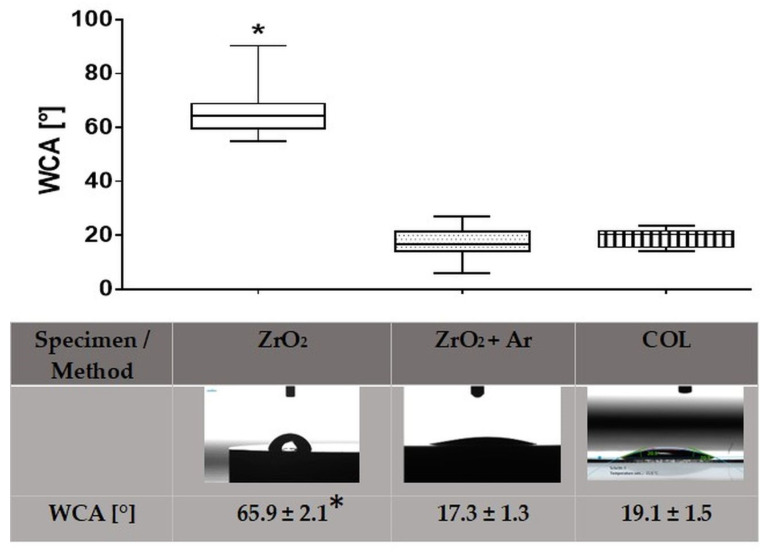
Wettability of specimen surfaces: zirconia untreated (ZrO_2_), after Ar plasma activation (ZrO_2_ + Ar), and glass with collagen coating (COL). Note the significantly higher water contact angle (WCA) on ZrO_2_, which is still in the hydrophilic range. (Drop Shape Analyzer DSA25, Krüss; at least 4 drops, Kruskal–Wallis test post-tested with adjusted Mann–Whitney test, * *p* < 0.05; upper row: median ± IQR, lower row: mean ± s.e.m.).

The characterization of surface wettability is displayed in Figure 2. Plasma activation of the zirconia specimens resulted in a statistically significantly lower water contact angle (WCA) than the polished untreated surfaces (ZrO_2_ + Ar: 17° vs. ZrO_2_: 66°). Moreover, the collagen-coated glass indicated a small WCA of 19°.

**Figure 3:**
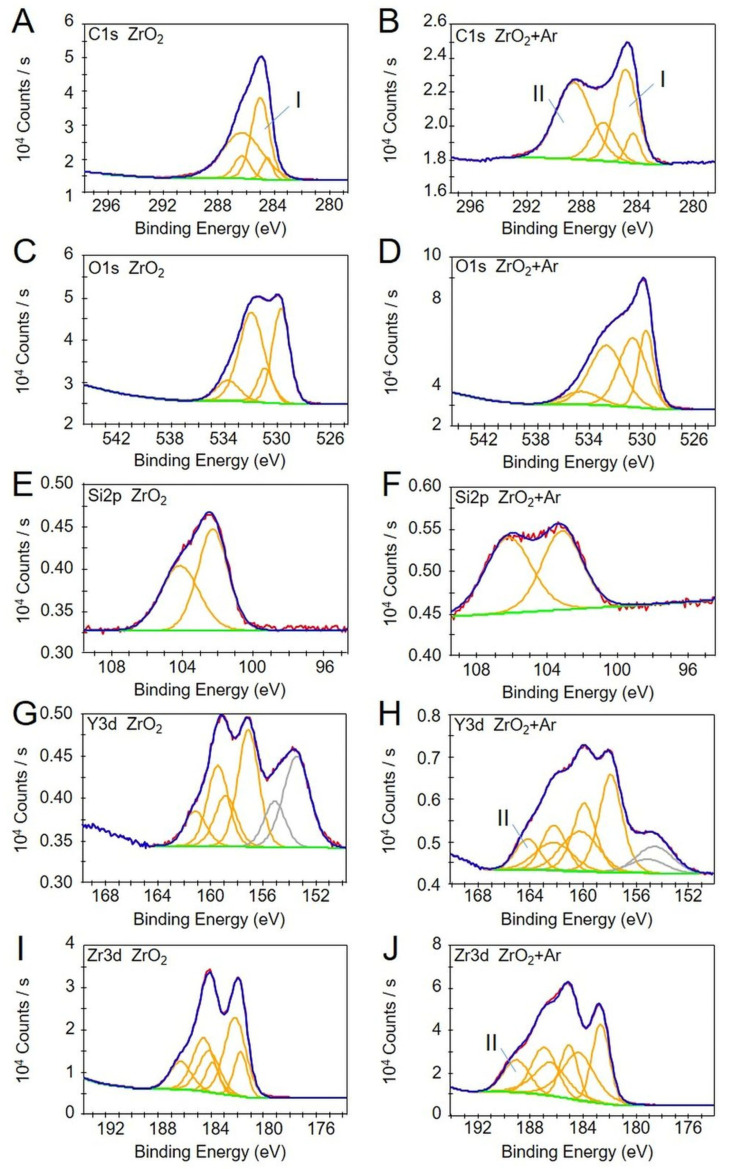
Detailed photoelectron spectra (XPS) for C1s (**A**,**B**), O1s (**C**,**D**), Si2p (**E**,**F**), Y3d (**G**,**H**), and Zr3d (**I**,**J**) of zirconia untreated (ZrO_2,_ left), and after Ar plasma activation (ZrO_2_ + Ar, right). Note the spectral contributions due to different chemical states (yellow), resulting envelopes (blue) and experimental data (red), and contributions due to Si in Y3d spectra (**G**,**H**) are marked in grey (K-Alpha XPS System).

Using X-ray photoelectron spectroscopy (XPS), differences between the untreated and plasma-activated samples can be observed in all the elements shown in Table 1. Thus, the areas under the specific signals change. There is a decrease for silicon (Si) and carbon (C) in ZrO_2_ + Ar compared to ZrO_2_ and an increase for yttrium (Y), zirconium (Zr), and oxygen (O). In the case of oxygen, an increase is observed preferentially at low binding energy, indicating the formation of metal oxides such as ZrO_2_ or Y_2_O_3_ (Figure 3C,D).

In addition, characteristic changes in spectral contributions are observed that can be attributed to specific bonding states. In the C1s spectra, a signal at higher binding energies is detected in the plasma-treated samples (Figure 3BII), accompanied by a decrease in carbon species at lower binding energies (C-C/C-H) (Figure 3AI,BI). This indicates the oxidation of carbon to carboxyl or carbonate species. In the detailed spectra of the elements yttrium and zirconium (Figure 3G–J), chemical states at higher binding energies are observed after plasma activation, indicating the formation of metal carbonates or silicates (Figure 3HII,JII).

### 3.2. Characterization of Human Gingival Fibroblasts (HGF-1)

#### 3.2.1. Growth over Time

**Figure 4:**
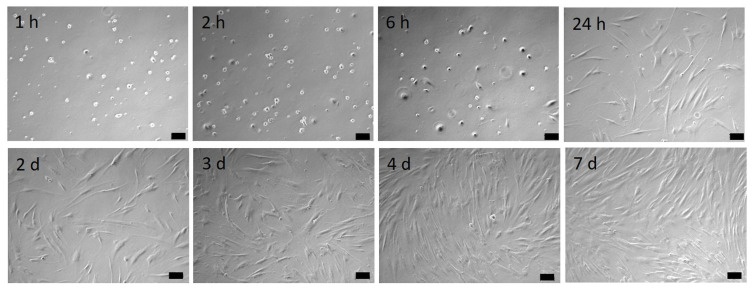
HGF-1 cell growth over time. Note that the spreading starts from 2 h cultivation, and after 24 h, the cells reached their maximum cell area. (Axiovert 40, Zeiss, 10× objective, scale bars = 100 µm).

To assess the adhesion, spreading, and growth of HGF-1 cells, morphology was documented over a 7 d period (Figure 4). It was observed that after 2 h of the adhesion phase, the cells start to spread out, and from 24 h onwards, they develop their typical cell shape of elongated spindle-shaped phenotype. After 7 d, a confluent cell layer could be detected.

#### 3.2.2. Expression of Adhesion Receptors

**Figure 5:**
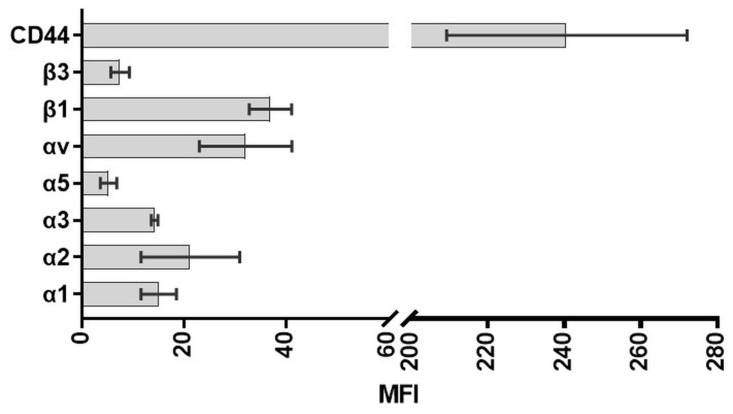
Expression of adhesion receptors in HGF-1 gingival fibroblasts. Note the strong expression of the hyaluronic acid receptor CD44. (FACSCalibur, BD Biosciences; mean fluorescence intensity MFI; n = 3, with 10,000 cells per approach).

The expression of a series of integrin receptor subunits and hyaluronan receptor CD44 of HGF-1 cells was characterized by flow cytometry after 24 h (Figure 5). The hyaluronan receptor CD 44 was distinctly expressed. The expression profile of integrin receptors indicated that the subunits αv and β1 were most accessible, whereas the integrin subunits such as α5 and β3 were weakly expressed in gingival fibroblasts. In the combination α2β1, this integrin receptor binds mainly to collagen type I.

#### 3.2.3. Expression of Adenosine 5′-Triphosphate (ATP) Receptors

**Figure 6:**
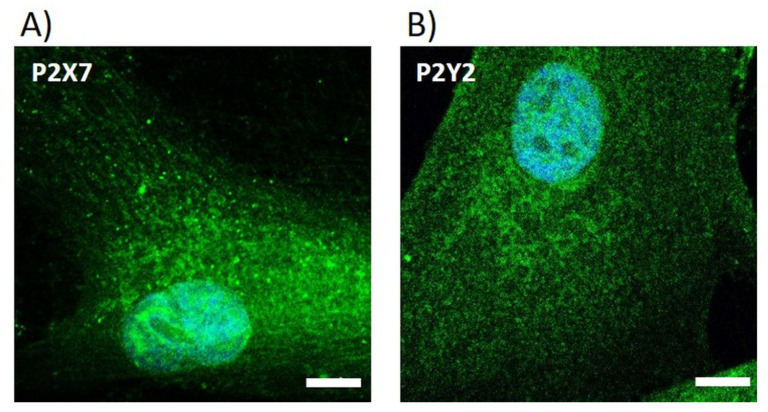
Expression of ATP receptors in HGF-1 gingival fibroblasts. Fluorescence images of ATP receptor type P2X7 (**A**) and P2Y2 (**B**) (green, cell nucleus in blue). (LSM780, Carl Zeiss; 63× oil objective, scale bars = 10 μm).

In the pre-study, it should also be demonstrated whether HGF-1 gingival fibroblasts are able to respond to ATP for intracellular calcium ion (Ca^2+^) mobilization. Therefore, the expression of ATP receptors was assessed (Figure 6).

ATP activates purinergic receptors: the ionotropic P2X receptors and the metabotropic P2Y receptors. P2Y2, as an ATP-sensitive G protein-coupled receptor, activates the phospholipase C pathway after binding ATP, which induces cells to mobilize their intracellular Ca^2+^ stores from the endoplasmic reticulum, leading to an increase in cytoplasmic Ca^2+^ levels. Fluorescence images of receptor types P2X7 (Figure 6A) and P2Y2 (Figure 6B) indicated the presence of these ATP receptors in HGF-1 fibroblasts.

### 3.3. Impact of Ar Plasma Activation on First Cell Response after 2 h

**Figure 7:**
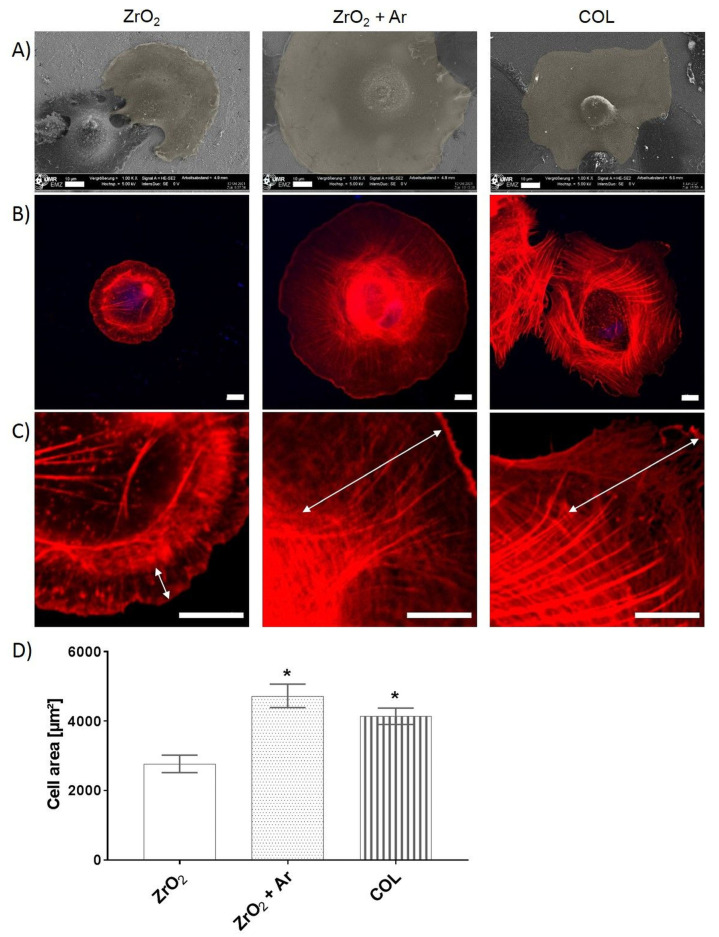
Growth of HGF-1 gingival fibroblasts on specimens after 2 h cultivation: (**A**) Morphology: although cells on pure ZrO_2_ start to spread out well after only 2 h, the increase in cell area on ZrO_2_ + Ar is obvious (FE-SEM Merlin VP compact, scale bars = 10 µm); (**B**) Organization of the actin cytoskeleton: long fine filament in the lamellipodia region of cells on ZrO_2_ + Ar, and COL; (**C**) Magnified images of actin, zoom 4: strong development of lamellipodia projections on the leading edge of the cell (see arrow), (LSM780, Carl Zeiss; 63× oil objective, scale bars = 10 μm); (**D**) Cell area: highest on Ar plasma-activated zirconia (ZrO_2_ + Ar) (ImageJ; 40 cells of actin images, mean ± s.e.m., Kruskal–Wallis test post-tested with adjusted Mann–Whitney test, * *p* < 0.05 compared to ZrO_2_).

To detect the impact of plasma activation, the spreading and morphology of HGF-1 gingival fibroblasts were determined by microscopy after 2 h cultivation on specimens. In Figure 7A, the FE-SEM images indicated a difference in cell spreading. On untreated zirconia (ZrO_2_), HGF-1 cells spread slowly and were still rounded in cell shape. The HGF-1 cells on plasma-activated zirconia (ZrO_2_ + Ar) exhibited the most pronounced spread (~1.7-fold to ZrO_2_) and attached to the surface with their whole cell body. The biological control with collagen coating (COL) also indicated improved spreading compared to ZrO_2_ (~1.5-fold), but not to the same extent as Ar plasma activation (Figure 7D) (ZrO_2_: 2647 µm ± 191 µm, ZrO_2_ + Ar: 4729 µm ± 339 µm, COL: 4141 µm ± 251 µm; mean ± s.e.m). The confocal microscopy images (Figure 7B,C) revealed that the cells started to form filaments at the leading edges. These lamellipodia are particularly prominent in cells on ZrO_2_ + Ar with numerous fine actin filaments. In contrast, the cells on ZrO_2_ were less spread, and rather cortical filaments were detectable around the cell, indicating the onset of adhesion and spreading.

### 3.4. Impact of Ar Plasma Activation on Cell Phenotype after 24 h

**Figure 8:**
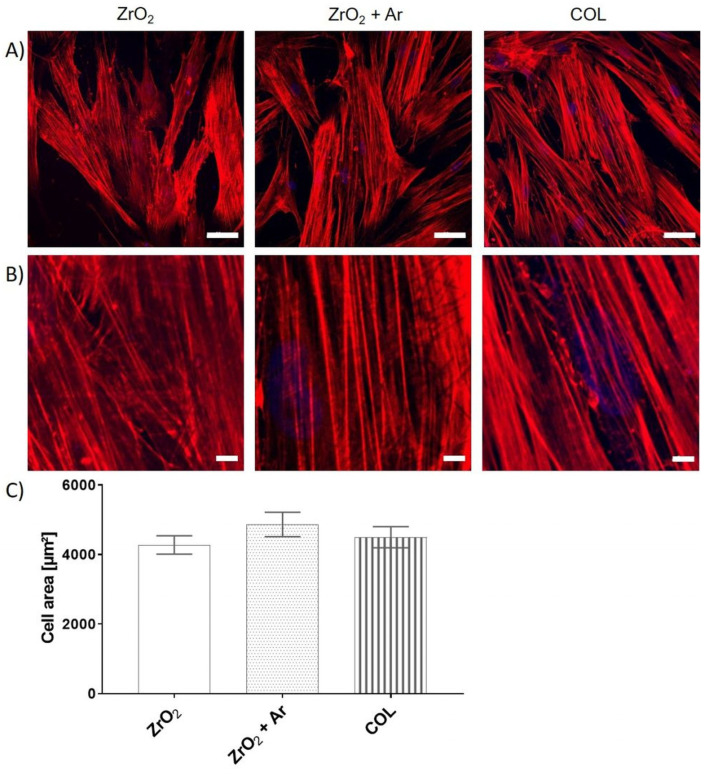
Growth of HGF-1 gingival fibroblasts on specimens after 24 h cultivation: (**A**) Organization of the actin cytoskeleton (scale bars = 50 µm) (**B**) Magnified view, Zoom 4 (scale bars = 5 µm) (Actin: red, cell nucleus: blue; LSM780, Carl Zeiss; 40× water objective), and (**C**) Cell area (ImageJ; 40 cells of actin images, mean ± s.e.m., Kruskal–Wallis test post-tested with adjusted Mann–Whitney test, n.s.). Note that after 24 h, the cell area is approximately the same on each specimen, but stronger actin filaments are detectable on Ar plasma-activated zirconia (ZrO_2_ + Ar) and collagen-coated glass (COL) compared to untreated zirconia (ZrO_2_).

After 24 h growth on specimens, the actin cytoskeleton in the cells on plasma-activated zirconia (ZrO_2_ + Ar) and collagen (COL) both appeared in well-organized strong stress fibers (Figure 8A,B), which span through the entire cell body. Cells on untreated polished zirconia (ZrO_2_) could also arrange well-organized long filaments, but the filaments were thinner compared to the treated surfaces (Figure 8B). The cell area of HGF-1 cells was not different on all specimens after 24 h (ZrO_2_: 4279 µm ± 265 µm, ZrO_2_ + Ar: 4868 µm ± 352 µm, COL: 4498 µm ± 301 µm; mean ± s.e.m) (Figure 8C).

### 3.5. Impact of Ar Plasma Activation on Calcium Ion (Ca^2+^) Mobilization

**Figure 9:**
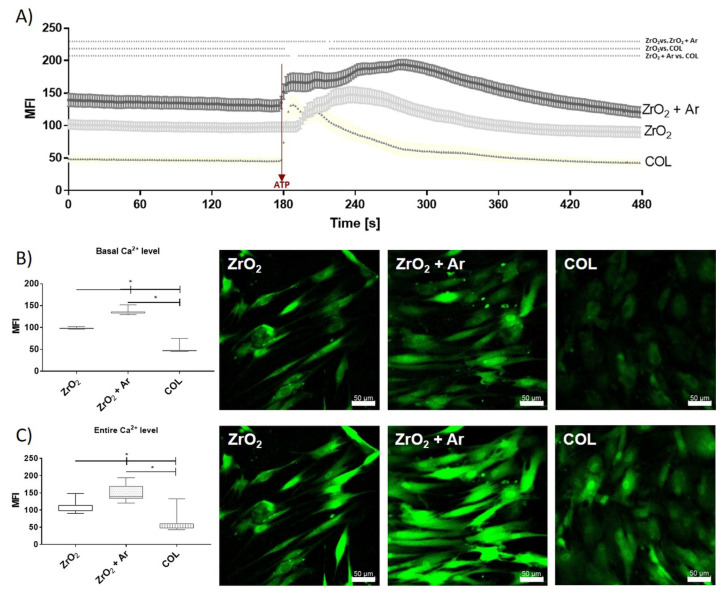
Intracellular calcium ion (Ca^2+^) mobilization in HGF-1 gingival fibroblasts on specimens after 24 h cultivation. (**A**) Time-dependent Ca^2+^ signals of Fluo-3-stained cells, with ATP addition after 180 s (arrow). (Calcium signal intensity, mean ± s.e.m., n = 30 cells from 3 approaches, * *p* < 0.05, Kruskal–Wallis test post-tested with adjusted Mann–Whitney test). (**B**) The basal level of intracellular Ca^2+^ (0–178 s; median ± IQR, n = 3, Kruskal–Wallis test post-tested with adjusted Mann–Whitney test, * *p* < 0.05), and representative images at the beginning (time point: 2 s; green: Ca^2+^ signal, scale bars = 50 µm). (**C**) The entire calcium level over time of 480 s (0–480 s; median ± IQR, n = 3 independent experiments à 10 cells, Kruskal–Wallis test post-tested with adjusted Mann–Whitney test, * *p* < 0.05), and representative images after ATP stimulation (time point 200 s; green: Ca^2+^ signal, scale bars = 50 µm). Note the significantly higher Ca^2+^ signals of the basal level as well as after ATP stimulation in cells on Ar plasma-activated zirconia (ZrO_2_ + Ar) compared to both untreated zirconia (ZrO_2_) and COL. (LSM 780, 40× water objective, 240 cycles every 2 s).

Intracellular Ca^2+^ signaling plays a crucial role in cellular interactions. In order to confirm the impact of plasma activation on cell signaling, the fluorescence measurements of vital Fluo-3-stained HGF-1 gingival fibroblasts were recorded via confocal microscopy after 24 h cultivation (Figure 9). During a defined period of 8 min, the basal Ca^2+^ signal was first recorded, and then, after stimulation with adenosine 5′-triphosphate (ATP), the Ca^2+^ was mobilized from the stores. Subsequently, defined areas of 10 cells were analyzed. Figure 5 shows the mean fluorescence intensity (MFI) time course over 240 cycles (á 2 s). HGF-1 cells on the plasma-activated zirconia (ZrO_2_ + Ar) exhibited a significant basal activity of Ca^2+^ compared with both controls (ZrO_2_, COL). Interestingly, the significantly lowest basal Ca^2+^ level was detected for fibroblasts on the COL surface compared to both zirconia surfaces (Figure 9B). The release of Ca^2+^ into the cytoplasm after ATP stimulation, as well as the entire Ca^2+^ signal over time, also revealed a significant increase in cells on ZrO_2_ + Ar compared to ZrO_2_ and COL (Figure 9C).

## 4. Discussion

Zirconia ceramics (ZrO_2_) are used for oral implants due to their esthetic and biocompatible performance [2,4,5,6,7,9,28]. A possible modern promising dental treatment that positively influences soft tissue integration and osteogenesis is a surface modification with physical plasma [10,11,18,21,22,25,31,35,39,40,47,48,49]. To analyze the impact of atmospheric pressure Ar plasma (kINPen^®^09) on the zirconia surface properties and, subsequently, on the cell behavior, we worked with polished specimens. We focused on how active cells behave on the surface and observed the calcium ion (Ca^2+^) signaling in human gingival fibroblasts (HGF-1).

Surface modifications with atmospheric plasma can improve the surface properties of titanium (Ti) and ZrO_2_ implant materials in terms of wettability [5,21,31,40,50], surface energy [51,52], and surface chemistry without affecting the surface structure (neither roughness parameters nor microstructure) [10,28,31,35,41,53,54,55]. Our study also showed no detectable changes in microstructure after a 1 min treatment with Ar plasma. Others, such as Noro et al. [21] demonstrated a change in the surface structure after 10 min treatment with an O_2_ plasma surface modification device, which could be due to the appropriate settings of the device used.

In several studies, it has been shown by XPS that Ti and ZrO_2_ surfaces treated with atmospheric pressure plasma can decrease the number of carbon residues such as polycarboxylate and carboxyl groups, thus significantly increasing the content of the other elements such as Ti and Zr, or the oxygen (O) content (oxide layers) of the surfaces [21,31,41,48,51,52,53,56]. Similarly, we observed a decrease in the carbon signal, which indicates an ablation of organic deposits, as shown by other groups [5,21,35,39]. It is also true for titanium-based biomaterials [51,52]. The appearance of silicon may have, according to the authors, several possible causes. Similar to organic deposits, they may originate from silicone deposits. It probably derives from the kINPen plasma device caused by its quartz capillary. During the plasma treatment, the implantation of silicon onto the ceramic surface may occur. The activation and oxidation of the organic and the silicon deposits follow. It converts a large portion of the carbon compounds to carbonate and the silicon compounds to silicate [57], which can react with activated sites of the elements yttrium and zirconium. It would lead to the formation of the corresponding yttrium carbonates, yttrium silicates, and zirconium silicates, supported by the formation of chemical states with higher electron binding energies in zirconium. To the same extent, the silicon signal changes accordingly [57], indicating the presence of silicate species [58].

Nevertheless, the silicon area does not decrease in the direction of complete material removal. Both possible causes for the appearance of the silicon signals can lead to the observed effects but cannot be separated at this point. The oxygen signal increase is expected and can be attributed to the reaction of the activated sites of yttrium and zirconium with atmospheric oxygen.

The strong hydrophilicity of the surface after plasma activation is mainly associated with an increased oxide layer, the introduction of hydroxyl groups, and the production of reactive oxygen species [21,53,56]. The surface potential can be converted from electronegative to electropositive. The study by Foest et al. [59] has shown that mixtures of noble gases such as Ar are suitable as metastable noble gas species (carriers of significant amounts of energy), leading to the formation of reactive oxygen species via energy transfer reactions, resulting in increased levels of surface reactivity and energy. Ionization of atoms and molecules occurs only within the first 20 µm [11].

It is plausible that treatment of the material with atmospheric plasma leads to the removal of surface carbon contamination and thus increases the wettability of the implant surface, increases protein adsorption, and subsequently promotes cell adhesion and crosstalk [10,21,25,31,35,39,40,41,47,51,54,56]. Cell adhesion is mediated by the amount and conformation to extracellular matrix molecules (such as fibronectin, collagen, or laminin) that are spontaneously adsorbed to surfaces by blood, other body fluids, and culture media [47]. Moderately hydrophilic and positively charged surfaces favor cellular adhesion levels [32,43,60,61]. Cell adhesion, a crucial phase of cell–material interaction [44], subsequently regulates cells´ proliferation and differentiation activity [62]. The increased wettability and surface free energy (increase in both polar and dispersive components [20]) demonstrated in our study could represent critical factors that ultimately influence the cellular response.

HGFs are reported to be the most abundant cells in the gingiva that produce an extracellular matrix for wound healing and regeneration after dental implantation [13]. The practical attachment of HGFs to the surfaces of implant materials plays a crucial role in soft tissue integration [11,12,28,63] and is critical for implant success. Therefore, this study investigated the cell attachment, morphology, viability, and signaling of HGF-1 cells on 1 min ZrO_2_ + Ar-activated materials compared to untreated ZrO_2_ and the biological control COL. The results of the present study suggest that the ZrO_2_ + Ar specimens may promote early attachment of soft tissue cells.

In the work of Hersel et al. [64], it is reported that attachment, spreading, cytoskeleton development, and cell-matrix adhesion formation are a cascade of events that occur during cells’ occupation of biomaterials We detected an increased expression of the adhesion receptor CD44 and moderate expression of integrin subunits β_1_ and α_v_ in HGF-1 fibroblasts. Adhesion receptors such as integrins act as mechanotransducers for extracellular signals and may enable subsequent signal transduction via the organization of the associated actin cytoskeleton [36,65,66]. Therefore, cell attachment, actin organization, and Ca^2+^ signaling are important biomaterial cytocompatibility indicators [42,43]. Due to plasma activation, in this study, we demonstrated improved adhesion and spreading and enhanced actin fibers in HGFs on the Ar plasma samples compared to untreated ZrO_2_. After only 2 h of adhesion on ZrO_2_ + Ar and COL, enhanced lamellipodia formation was demonstrated. Lamellipodia are the actual motor that pulls the cell forward during the process of cell migration and are considered an indicator of enhanced migration [67].

The finding of improved cell attachment is consistent with other plasma studies [10,28,35,47]; e.g., the effect of O_2_ plasma functionalization of titanium resulted in morphological changes concerning actin stress fiber and vinculin contact formation in HGF-1 cells [28].

The organization of the cytoskeleton and the formation of a cytoskeletal signaling complex subsequently affect intracellular Ca^2+^ mobilization [42]. Changes in cytosolic free Ca^2+^ regulate many cellular functions [68]. We showed that HGF-1 cells on ZrO_2_ + Ar specimens had a significantly increased basal calcium signal and increased the capacity to mobilize intracellular Ca^2+^ after adenosine-50 triphosphate (ATP) addition compared to ZrO_2_ and COL. Increased Ca^2+^-mobilization on COL would also have been expected, as it has been described that arginyl-glycyl-aspartic acid (RGD)-modified surfaces can trigger Ca^2+^ mobilization through integrin-mediated “outside-in” signal transduction during cell adhesion [69]. However, our study suggested that COL does not show increased Ca^2+^ signaling despite hydrophilic properties. Gruening et al. [32] also demonstrated reduced Ca^2+^ dynamics in MG-63 osteoblasts on COL-coated Ti surfaces compared to a plasma polymerized amino group containing nanolayer. A short-term, repetitive increase in free Ca^2+^ in the cytoplasm may be due to ATP [42,68,70], which, in turn, activates a class of receptors called purinergic receptors: the P2Y receptors (a superfamily of G-protein-coupled receptors) and the P2X receptors ligand-gated ion channels) [32,71,72]. In HGF-1 cells, we detected two types of ATP receptors. P2Y receptors, coupled to phospholipase C, mediate the action of extracellular nucleotides such as ATP, thereby mediating the increase in inositol triphosphate (IP3) and thus the release of Ca^2+^ from its intracellular stores—the endoplasmic reticulum. Therefore, the release of Ca^2+^ from its intracellular stores was given by ATP stimulation in HGF-1 cells.

The observed increased Ca^2+^ mobilization of ZrO_2_ + Ar specimens correlates well with the results of the enhanced actin cytoskeleton and improved cell growth of HGF-1 cells in the present study. Moreover, studies with osteoblast-like cells showed that viability was significantly increased on Ar plasma-treated microstructured Ti surfaces compared to the untreated control [31,40].

Some studies demonstrated improved proliferation and viability of MC3T3-E1 cells with O_2_ plasma-treated ZrO_2_ in direct comparison with Ar plasma-treated samples [35,41]. They would prefer the O_2_ plasma treatment. Since the kINPen can only be ignited with Ar-gas, a direct comparison with O_2_ plasma is impossible in our study. However, it would be interesting to see if another source could enhance the benefit of cell behavior. It is also possible that the effects on cell behavior are enhanced with more extended time intervals (>1 min).

The heterogeneity of the experimental conditions in the other studies (i.e., different cell types, diverse materials, plasma instruments and sources exposure times, Ar plasma systems, measured times, and protocols used) makes a direct comparison of the results difficult. A limitation of the study is the small sample size, which increases the margin of error. Nevertheless, a trend toward the effects of atmospheric pressure Ar plasma was observed here, providing a scientific basis for calculating an appropriate sample size to support further studies. However, the whole implant shape should not influence the outcome of the cell response because cells grow two-dimensionally on the surface of implants, which is the same situation as in our in vitro approach. Cells directly feel the surface characteristics solely beneath their basal attachment side in this nano–micrometer range. The implant design is in the mm range, and cells cannot react to mm range architecture. In this concern, we found out earlier that the movement (in nm per min) of vinculin contacts in GFP-vinculin transfected cells was the same on all different titanium roughnesses tested, e.g., polished, machined, glass-blasted, and corundum-blasted. This finding was astonishing because we measured roughness average values (Ra) of 0.19, 0.54, 1.22, and 6.07 µm, respectively. The roughness was more than 30 times higher from polished to corundum-blasted Ti, but the movement, vinculin length, and number were the same [73], as was also the β1-integrin expression.

The observed benefits of atmospheric plasma on wettability and, subsequently, the effects on cell adhesion should be confirmed in further in vitro studies (differentiation markers, markers such as cytokines and chemokines, and apoptotic factors) and in vivo studies to clarify the specific cellular context. More prolonged periods should also be a focus to find out whether there is only a temporary effect and to change the positive fibroblast response in the long term [28]. For dental applications, the storage time of Ar-activated samples is essential. How long is the duration of surface activation or the support for cells? This will be one focus of future studies.

## 5. Conclusions

Our data demonstrated the complex interactions between soft tissue cells and argon (Ar) plasma-activated zirconia (ZrO_2_ + Ar). Ar plasma activation of ZrO_2_ surfaces improved their hydrophilicity and promoted the early response of human gingival fibroblasts (HGF-1). In addition to enhanced spreading, improved actin organization and calcium ion signaling of HGF-1 were detectable on ZrO_2_ + Ar specimens demonstrating higher activity of the cells. Our results suggest that atmospheric pressure plasma treatment effectively improves soft tissue cell contact to achieve rapid and dense soft tissue sealing on zirconia implant abutments.

## Figures and Tables

**Table 1:** Element composition of sample surfaces ZrO_2_ (untreated) and ZrO_2_ + Ar (after plasma activation) as analyzed by X-ray Photoelectron Spectroscopy (XPS). (cps: counts per second).

Element	Peak Area	ZrO_2_	ZrO_2_ + Ar
**Si2p**	absolute (cps)	4704	3021
	relative (%)	1.59	0.62
**Zr3d**	absolute (cps)	71,442	185,766
	relative (%)	24.08	37.94
**Y3d**	absolute (cps)	4019	11,071
	relative (%)	1.35	2.26
**O1s**	absolute (cps)	111,786	255,777
	relative (%)	37.67	52.24
**C1s**	absolute (cps)	104,768	34,021
	relative (%)	35.31	6.95

## Data Availability

The data sets generated during and/or analyzed during the current study are available from the corresponding author on reasonable request. The data set is stored on the local Rostock University Medical Center (UMR) server.
